# Outcomes of patients with end stage kidney disease on dialysis with COVID-19 in Abu Dhabi, United Arab Emirates; from PCR to antibody 

**DOI:** 10.1186/s12882-021-02378-y

**Published:** 2021-05-26

**Authors:** Wasim Ahmed, Ali Abdul Kareem Al Obaidli, Princy Joseph, Edward R. Smith, Ayaz Ahmad Khan, Siddiq Anwar, Thangavelu Chandrasekar, Ayman Kamal Al Madani, Hormazdiar Dara Dastoor, Imran Zahid, Freddie Agbayani Costales, Yousef Abdul Rahim Boobes, Fatima Al Kindi, Salah Eldin Khalil Issa, Mohamed H. Hassan, Abraham George, Stephen Geoffrey Holt

**Affiliations:** 1 SEHA Kidney Care, PO BOX 92900, Abu Dhabi, United Arab Emirates; 2 University of Melbourne, Melbourne, Australia; 3 Sheikh Shakhbout Medical City, Abu Dhabi, UAE; 4 Tawam Hospital, Al Ain, UAE; 5 Madinat Zayed Hospital , Abu Dhabi, UAE; 6 Sheikh Khalifa Medical City , Abu Dhabi, UAE

**Keywords:** COVID-19, End stage kidney disease, Dialysis, IgG antibody, Mortality, Screening

## Abstract

**Background:**

Individuals with end-stage kidney disease (ESKD) on dialysis are vulnerable to contracting COVID-19 infection, with mortality as high as 31 % in this group. Population demographics in the UAE are dissimilar to many other countries and data on antibody responses to COVID-19 is also limited. The objective of this study was to describe the characteristics of patients who developed COVID-19, the impact of the screening strategy, and to assess the antibody response to a subset of dialysis patients.

**Methods:**

We retrospectively examined the outcomes of COVID19 infection in all our haemodialysis patients, who were tested regularly for COVID 19, whether symptomatic or asymptomatic. In addition, IgG antibody serology was also performed to assess response to COVID-19 in a subset of patients.

**Results:**

152 (13 %) of 1180 dialysis patients developed COVID-19 during the study period from 1st of March to the 1st of July 2020. Of these 81 % were male, average age of 52​ years and 95 % were on in-centre haemodialysis. Family and community contact was most likely source of infection in most patients. Fever (49 %) and cough (48 %) were the most common presenting symptoms, when present. Comorbidities in infected individuals included hypertension (93 %), diabetes (49 %), ischaemic heart disease (30 %). The majority (68 %) developed mild disease, whilst 13 % required critical care. Combinations of drugs including hydroxychloroquine, favipiravir, lopinavir, ritonavir, camostat, tocilizumab and steroids were used based on local guidelines. The median time to viral clearance defined by two negative PCR tests was 15 days [IQR 6–25]. Overall mortality in our cohort was 9.2 %, but ICU mortality was 65 %. COVID-19 IgG antibody serology was performed in a subset (*n* = 87) but 26 % of PCR positive patients (*n* = 23) did not develop a significant antibody response.

**Conclusions:**

Our study reports a lower mortality in this patient group compared with many published series. Asymptomatic PCR positivity was present in 40 %. Rapid isolation of positive patients may have contributed to the relative lack of spread of COVID-19 within our dialysis units. The lack of antibody response in a few patients is concerning.

## Introduction

The outbreak of acute pneumonia that emerged in Wuhan, China, in December 2019 [1] led to a pandemic classified by the World Health Organization as coronavirus disease 2019 (COVID-19) [2].

Clinical presentation of COVID-19 disease is variable, from an asymptomatic or mild disease (up to 80 %), to severe life-threatening illness with pneumonia (approximately 15 %). A minority of patients develop respiratory distress that mandates ventilatory support in the intensive care unit (ICU; 3–5 %). In some cases, an exuberant immune response to the virus can trigger an intense inflammatory reaction characterized by the presence of high levels of IL1- β, IL-6, TNF and other inflammatory chemokines like C-C motif chemokine ligands (CCL)-2,3 and 5 [3] (the so called ‘cytokine storm’). Such an inflammatory response may result in increasing pneumonia, myocarditis and renal impairment, which may be fatal. Mortality estimates in the general population range from 1.4 to 8 % [3–5]. According to initial data, factors associated with a poor prognosis include advanced age, male sex, and previous comorbidity, particularly cardiovascular events, diabetes mellitus, chronic obstructive pulmonary disease, or a history of cancer [6–8]. Recent data suggests CKD to be an independent predictor of severe disease [9, 10]. Analysis of 17 million records in the UK suggests that the highest mortality risks included CKD, Dialysis and organ transplantation (OpenSAFELY). The risks of CKD 3/4 exceed even diabetes or chronic heart disease [9, 11].

Patients with end stage renal disease (ESRD) on haemodialysis (HD) or peritoneal dialysis (PD) may be at higher risk due to their relative immune-incompetence, since uraemia is associated with impaired leucocyte function [11]. In addition, many patients have other co-morbidities like diabetes, hypertension and cardiovascular disease which further increases risk from COVID-19. Details of HD patients with Covid-19 were first reported in China in March 2020 [12], and since then further case series have been published with variable mortality rates.

Our service provides dialysis treatment across multiple sites in the Emirate of Abu Dhabi, UAE and is the largest dialysis provider in the country. We identified our first patient with COVID-19 in the middle of March 2020. Whilst we were proactive in identifying and isolating patients with and without symptoms, and we aimed to protect patients within dialysis units.

The objective of this study was to describe the characteristics of patients on dialysis who developed COVID-19 RT-PCR positivity, the outcomes of our screening and contact tracing strategy and to assess antibody response to COVID-19 in a subset of RT-PCR positive patients.

## Materials and methods

### Study design and participants

During the first phase of the pandemic, nasal swab RT-PCR testing of all patients, whether symptomatic or asymptomatic, was performed routinely every 14 days during the peak of the outbreak in line with local guidelines for identifying and containing the spread of COVID-19 in our dialysis units. Symptomatic patients or with close contacts with individuals known to have COVID-19 were immediately isolated and RT-PCR swabs were taken. Positive patients were reviewed in the hospital and then either admitted or isolated at home or hotels until 14 days after two negative PCR tests, taken at least 24 h apart. All positive patients had contact tracing to discover the most likely infective contact. Where we could establish a potential contact with a known positive within our facility (i.e. dialysing in the same bay or wing), we assumed that they acquired the virus in our dialysis unit. Where no such contact history was detected, we assumed community transmission. PCR screening continued every 14 days after recovery. Clinical recovery and negativity on PCR testing were not necessarily linked. Data was collected retrospectively between 1st March to 1st July 2020 from all our units. We did not include those patients with acute kidney injury who dialysed in our dialysis centres as most of these were related to COVID infection. No Paediatric patients (< 18 years) developed COVID-19 disease or screened positive for the disease. Patients with CKD not on renal replacement therapy, renal transplant recipients and patients with acute kidney injury requiring dialysis were excluded from analysis. Patients admitted to the hospital received prophylactic anticoagulation unless absolutely contraindicated and the decision to full or not to give any anticoagulation was made by the physician in charge based on an individual risk assessment and local guidelines.

### Data collection

Data were collected proactively by the infection control team and extracted from local data and electronic medical records (Cerner Millennium). All data were reviewed and checked by the primary investigator and the data collection team. Risk and severity assessments were done by the admitting physician based on defined clinical and chest CT scan results criteria. All data were collected at the time of the admission and analysed retrospectively.

### RT-PCR and antibody testing

COVID-19 RT-PCR were performed using Cobas SARS-CoV 2 Test by Roche Diagnostics (Roche Molecular Systems, Branchburg, NJ, USA). From the COVID-19 PCR positive patients, a subset underwent antibody testing for COVID-19 IgG antibodies, and testing was performed using the Abbott Architect SARS-CoV-2 IgG *(*Abbott SARS*-*CoV*-*2 IgG, Abbott Diagnostics, Abbott Park, IL, USA*)* which detects anti-nucleocapsid protein [13]. These and other diagnostics tests had regular and routine quality control as expected within an accredited pathology network.

The institutional review board including an ethics and scientific committee approval was obtained to analyse the data anonymously (Abu Dhabi Department of Health’s COVID-19 Research Ethics Committee (CVDC-20-05/2020-8, https://www.doh.gov.ae/en/covid-19/Research%20Registry). The informed consent for the study was waived by the committee due to the retrospective nature of the study and that COVID swabs or antibody testing was considered part of routine care.

### Statistical analysis

Continuous variables are expressed as mean (standard deviation) or medians (with interquartile range). Normality was assessed by Shapiro-Wilke, D’Agostino & Pearson and Kolmogorov-Smirnov tests. For statistical analyses, a 2-tailed unpaired t-test was used for parametric continuous variables, for multiple comparisons ANOVA was used with appropriate corrections. For non-parametric data Kruskal-Wallis was used with Dunn’s correction. The association between variables was tested by Spearman rank co-coefficients. Logistic regression was used to determine odds ratios, and only significant univariate correlates were included in multivariate models. A *P*-value (alpha) of 0.05 or less was considered significant. Tests were performed using GraphPad Prism version 8.4.3 for Mac, GraphPad Software, San Diego, California USA, www.graphpad.com.

## Results

**Fig. 1:**
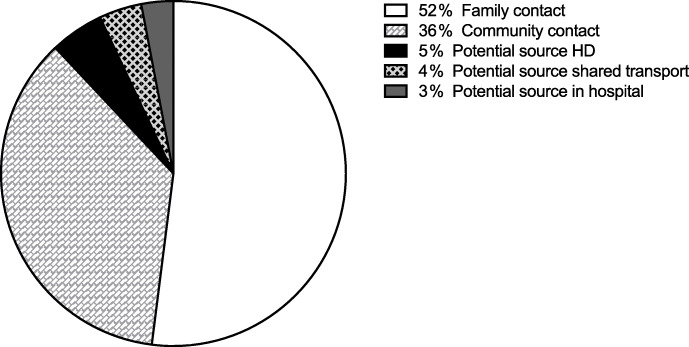
Source of acquiring COVID-19

During the study period of 4 months, 152 patients of our dialysis cohort developed a positive COVID-19 RT-PCR test, which represents ~ 13 % of our dialysis population at the start of the study period (*n* = 1180). Most of whom (81 %) were male and mostly dialysing in-central dialysis facilities (95 %), with 5 % on PD at home. This compares with approximately 61 % of our general dialysis population who are male with 7 % on PD. There were over 16 different nationalities represented in the COVID positive group. The age of COVID-19 PCR positive was normally distributed patients with a mean 52 ± 12 years (median 53 [IQR 44-60]) years and with a range of 20–85 years. Around two thirds (59 %) of patients had symptoms at the time of initial RT-PCR testing. Contact tracing was performed in all patients, and a COVID-19 positive close family contact was identified in ~ 52 % (79/152) of patients. We were especially interested in defining a link to index patients within our dialysis facilities to test the robustness of our PPE precautions. In 36 % (56/152), we could not connect the patient to any positive COVID-19 contacts within the dialysis unit. We found 5 % (7/152) who we traced to a potential contact as they may have dialysed in proximity to an index case (same bay or wing). A further 4 % (6/152) cases were linked to positive cases by undeclared shared transport to the dialysis unit, before we changed transport options for all patients to avoid any sharing. A final 3 % (4/152) have acquired COVID-19 during unrelated hospital admissions due to close contacts with positive cases in the hospital (Fig. 1).

Those patients whose presenting symptoms were recorded in the notes, reported fever and cough as the most common complaints (49 and 48 % respectively). Other symptoms reported by our patients included dyspnoea (16 %), myalgia (13 %), gastrointestinal symptoms (6 %) and loss of taste (1.3 %). Significant associations with outcome are given in Table 1. The most frequent comorbidities were hypertension (93 %), diabetes (49 %) and ischemic heart disease (30 %). Risk assessment was performed at admission and patients were categorized into mild which included asymptomatic cases (68 %), moderate (21 %) and severe (11 %) disease, based on clinical, laboratory and chest imaging findings. Around half of the patients had an abnormal chest imaging at admission.

**Table 1:** Clinical features of the dialysis patients who had positive COVID-PCR

	Total % (*n* = 152)	Alive % (*n* = 138)	Died % (*n* = 14)	*P*-value*
**Demographics**				
Age, y	52.4 (12.1)	51.2 (11.3)	64.1 (3.5)	0.002
Male Sex, %	81 (123)	81 (112)	79 (11)	ns
**Co-morbidities**				
Diabetes, % (n)	51 (78)	54 (75)	21 (3)	0.024
Cardiovascular disease, % (n)	70 (106)	72 (100)	43 (6)	0.032
Smoker, % (n)	43 (65)	41 (57)	57 (8)	ns
**Presenting symptoms**				
Fever, % (n)	49 (74)	44 (61)	93 (13)	< 0.001
Cough, % (n)	47 (72)	43 (60)	86 (12)	0.004
Dyspnoea, % (n)	16 (24)	12 (16)	57 (8)	< 0.001
Myalgia, % (n)	13 (19)	12 (17)	14 (2)	ns
GI symptoms, % (n)	6 (9)	4 (6)	21 (3)	0.038
**Disease severity**				
Mild, % (n)	68 (104)	75 (103)	7 (1)	ns
Moderate, % (n)	21 (32)	20 (28)	29 (4)	ns
Severe, % (n)	11 (16)	44 (7)	64 (9)	< 0.001
**Radiological findings**				
Abnormal chest x-ray, % (n)	45 (68)	40 (55)	93 (13)	< 0.001
Abnormal chest CT, % (n)	53 (80)	49 (67)	93 (13)	0.001

*P-value determined using two-sample Wilcoxon rank-sum (Mann-Whitney) test for continuous variables and Fisher's exact test for categorical variables

**Fig. 2:**
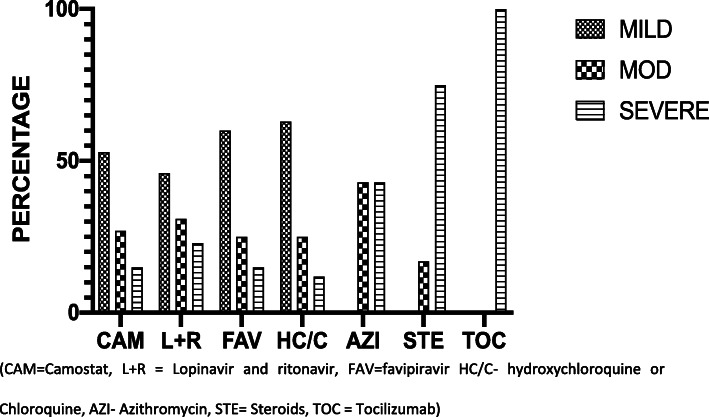
Percent of patients in mild moderate and severe categories receiving drugs therapies

Different pharmacological combinations were used (as recommended in the UAE national guideline and defined by disease severity), but treating physicians had flexibility to choose different drug regimens from the guidelines. The details of drugs used in mild, moderate and severe disease are given in Fig. 2.

ICU admission was required in 20 patients (13 %). 18 (90 %) of those needed mechanical ventilation and 14 (70 %) required continuous renal replacement therapy (as opposed to continuing with intermittent dialysis therapy) due to severe haemodynamic instability usually requiring inotropic support. Admission to the ICU for CKD patients came with a very grave prognosis with 13 dying in ICU (65 % mortality in this group). One patient recovered from his COVID disease and came out of ICU but died within the same admission of a cardiac problem, making the overall mortality in our population 9.2 % (14/152). The median time from positive PCR to death in this group was 14 days [IQR 4–23].

**Fig. 3:**
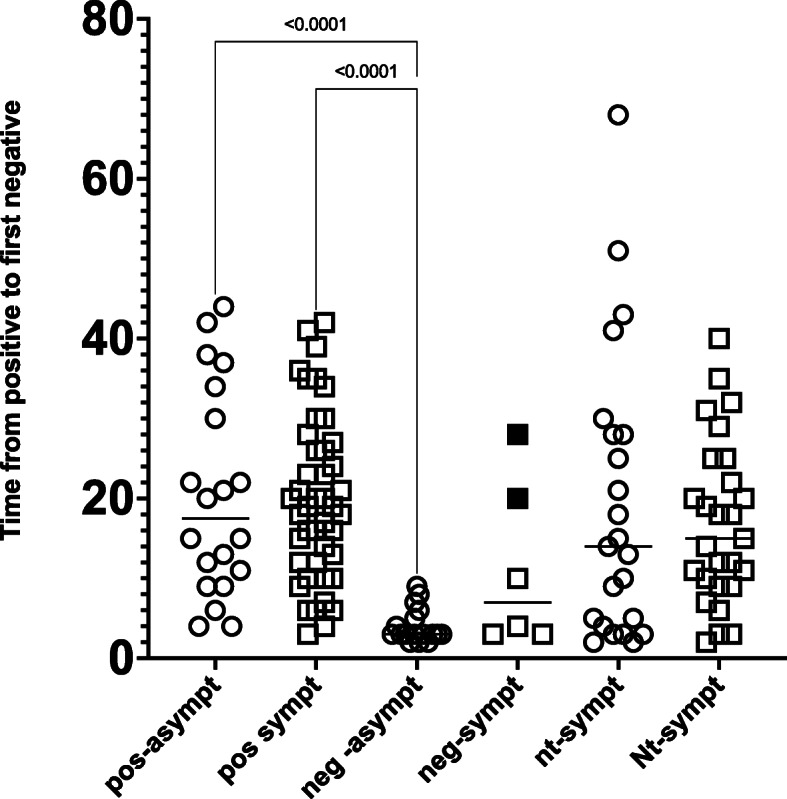
Days from COVID PCR Positive to the first of two negative COVID PCR tests in patients with and without detectable IgG antibodies and with and without symptoms at presentation. Circles – patients asymptomatic at time of first covid positivity. Squares – patients with symptoms at presentation. The two filled squares are patients who presented with good evidence of COVID disease but who did not develop an antibody response. Kruskal-Wallis tests with Dunn’s correction is shown between groups, apart from those shown, all other comparisons were not significant

A subset of PCR positive patients (*n* = 87) underwent COVID-19 nucleocapsid IgG antibody testing after a mean interval of 87 ± 16 days from the first positive PCR sample. Most (74 % 64/87) patients had positive IgG serology, with median time to testing of 85 ± 16 days. In these patients who were antibody positive, the median time to viral clearance was 19 days (IQR 11–28) (Fig. 3). In those not antibody tested this was 15 days ([IQR 7–26]). In contrast, those in whom an IgG antibody was not detected had much shorter times from positive to first negative PCR (median 3 days [IQR 3–7]), after testing after a mean of 92 ± 16 days. We found no difference in the median length of PCR positivity when we subdivided the antibody positive patients into those presenting with (median 19[IQR 12–26], mean 20 ± 13) and without symptoms (median 18[IQR 10–33], mean 20 ± 13), p = ns. Those with negative antibodies and without symptoms had a median time to negative of 3 days [IQR 3–6], whilst those with symptoms it was 7 days [IRQ 7–28]. In patients who were not tested for IgG antibody without symptoms median time to clearance was 14 days [IQR 4–28] whilst for those with symptoms 15 days [IQR 9–25]. Overall, the median positive time was 15 [IQR 7–26] days which was not significantly different to the IgG positive patients.

In the COVID-19 PCR positive patients we report no detectable antibodies in 26 % (23/87) of those tested. The majority of these (17/23, 74 %) were asymptomatic and mostly had a single positive PCR result, and we cannot exclude a false positive PCR in these patients. Six other interesting patients were those in whom the clinical presentations were consistent with COVID-19 disease and with a positive PCR test, but in whom antibodies did not develop. In this group, 4 had a single positive PCR tests, but two patients (Fig. 3 shaded points) had multiple positive PCR results respectively making it highly probable they had COVID-19 infection but did not develop antibodies. One such patient with polycystic disease was repeatedly tested, and although presented with moderate symptoms, had antibody testing at day 15, 16 (although disregarded in this analysis), and day 77, but all remained negative. The other patient who with renal disease of unknown origin also had multiple positive PCR’s and presented moderately symptomatic, but also failed to develop antibodies when tested 121 days after first positivity.

Following the completion of our study, we have become aware of one patient who became COVID PCR positive again 4 months after his initial positive PCR test. This patient had a single positive PCR with mild symptoms during the study but did not develop antibodies. He was recently found to have become positive again on screening and developed symptoms. No other patients who have had previously positive tests have retested positive after some months and none who have had demonstrable antibodies have ever retested PCR positive.

There was no significant difference between the groups in days to clearing the virus, except when comparing the negative asymptomatic group to the antibody positive group (Fig. 3).

## Discussion

 At the time of writing, the UAE had suffered a single wave of infection and current statistics from the pandemic (Worldometers) suggest that there have been 4,44,398 cases to date in a population of 9,940,597, giving an expected number of cases per million of 44,705. Patients with ESKD are immunocompromised, have high comorbidities and are among the most vulnerable populations to develop severe disease and death from COVID-19. Additional risk factors include difficulty to achieve social distancing for patients who need in-center haemodialysis and in some cases use of public transportation to get to dialysis units. Thus, we can be relatively confident that we identified most patients infected with COVID-19 in our dialysis cohort. It is possible that we missed asymptomatic patients who became transiently infected but cleared virus within 14 days.

Males were overrepresented in our COVID cohort (81 %) compared with our general dialysis cohort (~ 62 % Male). We are currently unable to explain why we seem to have an overrepresentation of males in the COVID-19 group, but other studies have also reported a male predominance and fatality rate [14, 15].

One strength of this study was the detailed contact tracing that occurred in our patients, and this pointed towards acquisition of virus mainly outside the dialysis units. This data that the strategies we had in place for protecting our patients appears in the main to be effective within the confines of our dialysis facilities. Furthermore, it gave us confidence to continue to dialyse COVID positive dialysis patients within the out-patient dialysis facility, albeit in isolation with level 3 PPE for 14 days after the last negative, before returning to the open dialysis bays. We also cohorted a number of patients based on a risk assessment of living conditions outside dialysis to try to keep patients as safe as possible as those in high density communal living were considered highest risk. PD patients were instructed on how to self-isolate. We were assisted in our COVID response by the support of the Abu Dhabi Dept of Health who made funding available, and the SEHA procurement, distribution and emergency coordination teams who ensured that we had no PPE shortages, and had dialysis facilities with enough isolation rooms.

Despite the high community transmission, it appears that such strategies were somewhat successful in protecting most of our patients from COVID-19, with a relatively lower percentage of patients within our centres developing COVID 19 compared to international comparators. A study from the UK Renal Association reported that London had the highest percentage of patients with COVID-19 (~ 19 %), with a range of 10–24 % among its dialysis centers [16]. The overall mortality in our population was also lower as compared to 14–31 % in China [17], United Kingdom 19 % [18], Turkey 20 % [19], Spain 23 % [5], Italy 28 % [20], and the United States 31 % [21]. A recent published study from French dialysis units (COVIDIAL) reported mortality of 24 % [22]. However, median age in this study was 77 (IQR 68–83) years and in those who died was 80 (IQR 72–88) years, with 66 % of those who died in this study were above the age of 75 years. The relatively low mortality within our cohort may reflect a relatively young median age of dialysis patients as older age definitely appears a risk factor for mortality (Table 1). Mortality among patients admitted to ICU requiring mechanical ventilation in our cohort was 65 %. Similar mortality rates of ~ 75 % in ICU patients have been reported [21].

There was a univariate association with smoking, and this may not be unexpected given the mode of mortality was mainly related to acute lung injury which would be no doubt affected by a smoking history. Symptoms predicting mortality were dyspnoea and gastrointestinal symptoms, which may again reflect the organs taking the brunt of the Covid-19 related injury. The COVIDIAL study reported higher mortality in patients who presented with dyspnoea (70 % versus 45 %; *P* = 0.027) [23]. The association of severe disease and CT changes are unsurprising, but the inverse association of comorbidity (diabetes and cardiovascular disease) may well be a type 1 error, related to low numbers. In other studies diabetic nephropathy was more common in patients who died (48 versus 32 %) and had a history of ischemic heart disease (61 versus 41 % *p* = 0.084), however, none of the specific comorbidities were associated with high risk of death [22].

Presence of COVID IgG antibodies in most PCR positive patients may give us hope that they have now protective immunity, at least temporarily and it will be interesting to explore how long lived such antibodies are. Some patients (*n* = 23) did not develop an antibody response (and thus presumably protective immunity) despite apparent infection. We also found a number of patients who were asymptomatic and had a PCR positive test (followed by 2 negatives and negative IgG) and we speculate that these were false positive PCR tests. Nevertheless, these patients were still isolated for 14 days after the initial PCR testing. Such antibody responses have been detected in ICU and non-ICU patients with COVID-19 [24]. 6 patients with symptomatic positive PCR tests had negative IgG. In 2 patients with good evidence of a COVID19 infection, we do not have a good explanation for this, and there was no history of immunosuppression in either. It is possible we missed the peak antibody titres and they had been transiently positive, but we did not test at the right time interval after infection. However, it suggests that at least some of our patients may have difficultly generating antibodies and hence a memory response to COVID disease, or that such a response is only detectable transiently. We acknowledge that in our antibody testing cohort we had a long interval between first positive and antibody testing, however optimal time to check antibodies is not known.

One recent study of SARS-CoV-2 IgG prevalence reported 40 % of patients with antibodies were either asymptomatic infection or had undetected disease by PCR testing. In this cohort however almost all (~ 98 %) of PCR-positive patients had detectable antibodies at a mean time of 34 ± 6.4 days after PCR testing [23]. However, they reported 22 % of their total study population contracted COVID-19 and also concluded that their strategy of not isolating patients who were asymptomatic, but antibody positive may have contributed to the spread of infection within their unit. Our study differed in that we performed RT-PCR on all of our dialysis cohort, symptomatic or asymptomatic and antibody on subset of positive PCR patients. We feel this was a better strategy in identifying any asymptomatic carriers, isolating them and preventing the spread of the infection within the dialysis unit and the community.

As multiple COVID-19 vaccines have been approved for use, we are hoping that dialysis patients will develop protective immunity but we are however cognisant of the fact that many vaccines, for example hepatitis B are relatively less effective in the dialysis population [25] than with those without renal impairment, so we are likely to need special seroconversion studies in this population. It is important that we collect data to understand how patients on dialysis and with renal transplants respond to standard vaccination schedules. Another study using lateral flow immunoassays (LFIAs) point of care testing, found 97.4 % of haemodialysis patients and 94.7 % of transplant recipients, tested positive for SARS-CoV-2 IgG antibody [26]. One of the limitations of LFIA is the potential to cross-react with the antigen used in test if patients had prior exposure to other beta-coronaviruses. It is of note that 15 % of patients in this study did not have detectable IgG antibodies when checked less than 21 days of having a positive PCR, however majority of these demonstrated antibodies on a second test after 21 days. It has been suggested that seroprevalence rates may vary depending on geographical location, the population being studied, and timing of analysis [27]. We anticipated measuring antibody in all our PCR positive patients or repeating the test, but this could not be performed due to operational reasons. Another notable result was that presenting symptoms could not be used as a reliable guide to antibody positivity, nor the length of COVID PCR persistence.

The presence of detectable PCR positivity in recuperating patients is well described [28] but the significance is unknown. It has been suggested that this may represent shedding of bits of replicating incompetent RNA fragments of the virus that have been processed by immune cells. The PCR test is incapable of distinguishing replicating competent virus from RNA fragments potentially excreted by viral destruction by immune mechanisms. We used this positive result to inform the isolation strategy, and these recovering positive patients remained in isolation. We reset the isolation clock based on these results.

Limitations of our study included the issues around assigning causality from retrospective and associative data. In addition, we were unable to assess use of ACE inhibitors, ARB, immunosuppressive medication, or statin usage in our cohort, along with lack of a full dataset on laboratory data, e.g. C-reactive protein, WBC count, Interleukin-6 (IL6), ferritin, LDH etc. However, correlation of these parameters and outcomes has already been published.

Additional speculative hypothesis that remains theoretical is that because of the mandatory and early ubiquitous mask use that we mandated within our facilities, and encouraged our patients to continue at home, may have reduced the inoculum of the virus for those patients that contracted COVID, potentially allowing for a milder disease [29], or possibly younger ages in our cohort.

## Conclusions

Patients with ESKD on dialysis are at high risk of contracting COVID-19 virus infection. At the time of completion of our study, the epidemic curve was on a downward trend in many countries, however, many countries are now facing a second or third wave. Our study has indicated an important finding of failure to generate a detectable antibody response in a number of patients. The significance of this finding may be relevant when we start vaccinating patients on dialysis, as seroconversion levels in this group may be lower. We have also shown that community transmission of COVID-19 remains the largest threat to our patients and staff, when an effective screening strategy, infection prevention and control measures are instituted within and encouraged outside of dialysis units.

## Data Availability

The datasets used and /or analyzed during the current study are available from the corresponding author on reasonable request.
